# Prevalence, awareness, treatment and control of hypertension in Guangxi Zhuang Autonomous Region

**DOI:** 10.1038/s41598-021-04735-1

**Published:** 2022-01-18

**Authors:** Guan Fan, Zhiyuan Jiang, Jianling Li, Liu Shi, Chun Gui, Rongjie Huang

**Affiliations:** 1 Department of Cardiology, The First Affiliated Hospital of Guangxi Medical University, No 6 Shuangyong Road, Qingxiu District, Nanning, Guangxi Zhuang Autonomous Region People’s Republic of China; 2 Guangxi Key Laboratory of Precision Medicine in Cardio-Cerebrovascular Diseases, No 6 Shuangyong Road, Qingxiu District, Nanning, Guangxi Zhuang Autonomous Region People’s Republic of China; 3 Guangxi Clinical Research Center for Cardio-Cerebrovascular Diseases, No 6 Shuangyong Road, Qingxiu District, Nanning, Guangxi Zhuang Autonomous Region People’s Republic of China

**Keywords:** Hypertension, Diagnosis, Disease prevention, Health care economics, Patient education, Public health

## Abstract

Hypertension (HTN) is getting more prevalent in China, but the HTN's status in Guangxi remains unclear. Our study started from 2013 to 2015 and was dedicated to better acknowledging the status of HTN in Guangxi. The study enrolled 17,100 residents aged ≥ 15 years across Guangxi, from 2013 to 2015, using a stratified multistage random sampling method. Parameters including blood pressure (BP), height, and weight were measured by validated devices. HTN was defined as the average systolic BP (SBP) ≥ 140 mm Hg and/or average diastolic BP (DBP) ≥ 90 mm Hg, or any usage of antihypertensive medications within two weeks. The awareness, treatment, and control were defined as a self-reported history of HTN, a self-reported current usage of antihypertensive medications, and a BP lower than 140/90 mm Hg, respectively. The age and sex-standardized prevalence, awareness, treatment, control rates of HTN for the population aged ≥ 15 years in Guangxi were 15.80%, 16.48%, 11.99%, 3.62%, respectively. Prevalence and control rates were the same for men and women (*P* > 0.05), while women’s awareness and treatment rates were higher than those of men (*P* < 0.05). Zhuang nationality had a higher prevalence than Han (23.50% vs. 20.35%, *P* < 0.001), while Han had higher awareness, treatment, control rates (37.39% vs. 31.22%, 30.59% vs. 22.37%, 8.99% vs. 4.55%, individually, *P* < 0.05). HTN was prevalent in Guangxi, while the awareness, treatment, control rates were adverse. Region-specific strategies to intervene in HTN were needed.

## Introduction

Hypertension (HTN) is one of the well-established modifiable risk factors for cardiovascular disease (CVD) and plays a critical role throughout the cerebrovascular disease and kidney disease progress^1–3^. Uncontrolled hypertension accounted for approximately 750,000 deaths due to CVD at 35 to 79 years of age in 2010 in China, and in 2017 the high systolic blood pressure (SBP) was one of the leading risk factors contributing to deaths and disability-adjusted life-years in China^4,5^.

The better management of HTN is a pivotal part of public health work. Strategies for the prevention and control of HTN must be established based on epidemiological characteristics of HTN among the local population. Guangxi Zhuang Autonomous Region is home to the Zhuang ethnic minority, China's largest minority group. Epidemiological features of HTN here had shown remarkable heterogeneities compared with other provinces, according to several previous studies^6,7^. Very few studies had presented the status of HTN in Guangxi, and epidemiological characteristics of HTN in Guangxi remain unclear. Therefore, studies about epidemiological features of HTN in Guangxi are warranted. In the current study, we investigated the status of HTN in the population aged 15 years or more of Guangxi, and several potential risk factors were detected as well, aiming to provide a peep at the basic epidemiological features of HTN and to shed light on the better control of it in Guangxi.

## Methods

Our study was a part of the China Hypertension Survey, 2012–2015, and the investigation started from 2013 to 2015.

### Survey participants

The design of the China Hypertension Survey study was published formerly^8^. In brief, based on the China Hypertension Survey Project protocol, a random sampling group was selected using a stratified and multistage random sampling method. A sample representing the general population aged ≥ 15 years from all 31 provinces in mainland China was obtained. All 31 provinces of mainland China were divided into urban and rural areas to select samples from, using a four-stage random sampling method. The first stage was to choose four urban areas and four rural areas in each province according to the probability proportional to size. In the second stage, two districts or two townships of urban areas or rural areas were selected using a simple random sampling method (SRS), and the third stage was to choose three communities or villages from these districts or townships separately. Then the final stage was to select a given number of participants from each of the 14 sex/age strata (men/women and aged 15 to 24 years, 25 to 34 years, 35 to 44 years, 45 to 54 years, 55 to 64 years, 65 to 74 years, ≥ 75 years) by SRS in the areas selected in the third stage. A total number of 18,000 residents (resided for 6 months or longer) from Baishou town and Sanhuang town, Yongfu; Luoman town and Sandu town, Liujiang; Pingyang town and Sanwu town, Laibin; Datang town and Fumian town, Yulin; Luoxiu town and Luobo town, Guiping; Xinjiang town and Baiji town, Yongning; Luwo town and Luobo town, Wuming; Wenfeng district and Jianshan town, Qinzhou; Bailin town and Fenghuang town Bama were expected to be involved. In the final, 17,100 were enrolled and finished the survey. Each participant was informed thoroughly of the survey's content, and written informed consent was obtained. The Ethics Committee of Fuwai Hospital (Beijing, China) and First Affiliated Hospital of Guangxi Medical University (Guangxi, China) approved the study. This study was strictly in accordance with the Declaration of Helsinki.

### Data collection

All staff members enrolled in the study were thoroughly trained to get a clear perception of the aim of the research and the specific tools or methods to be used. A standardized questionnaire developed by the coordinating center, Fuwai Hospital, was administered by trained staff to collect information on demographic characteristics and social-economic factors.

Height was measured to the nearest 0.1 cm without shoes by the standard right-angle device. Standard measurements for weight and body fat were determined by the Omron body composition monitor (V-body HBF-371, OMRON, Kyoto, Japan). Body Mass Index (BMI) was calculated as weight divided by the square of height (kilogram kg/m^2^).

Methods to measure BP were the same as our previous study^9^. Briefly, systolic blood pressure (SBP) and diastolic blood pressure (DBP) were standardly measured three times or more in the seated position with the OMRON Professional Portable Blood Pressure Monitor (HBP-1300, OMRON, Kyoto, Japan), after at least 5 min of rest. In 2% of the present samples, SBP and DBP were measured using both the OMRON device and a mercury sphygmomanometer (Yutu, Shanghai Medical Instruments Co., Ltd., Shanghai, China) for calibration. Measurements were repeated if there was a gap greater than 4 mm Hg between SBP or DBP values with the mercury sphygmomanometer or 10 mm Hg with the oscillometric BP monitor. The mean of the 3 closest values of SBP or DBP was used.

### Outcome definition

In accordance with the Chinese guidelines for the management of hypertension (2010), HTN was defined as SBP ≥ 140 mm Hg and/or DBP ≥ 90 mm Hg; or any usage of antihypertensive medications within two weeks. Pre-hypertension was defined as SBP among 120–139 mm Hg and/or DBP among 80–89 mm Hg without any usage of antihypertensive medications; Stage 1, 2, and 3 of HTN were defined as SBP among 140–159 mm Hg and/or DBP among 90–99 mm Hg, SBP among 160–179 mm Hg and/or DBP among 100–109 mm Hg, and SBP ≥ 180 mm Hg and/or DBP ≥ 110 mm Hg, individually^10^. Awareness of HTN was defined as a self-reported history of HTN diagnosed previously by the doctor, treatment of HTN was defined as a self-reported usage of any prescribed antihypertensive medications in the past two weeks, and control was defined as average SBP < 140 mm Hg and average DBP < 90 mm Hg. We also performed a post-hoc analysis using the criteria of the 2017 American College of Cardiology (ACC) /American Heart Association (AHA) High Blood Pressure Guideline, which defined HTN as SBP ≥ 130 mm Hg and/or DBP ≥ 80 mm Hg while control as SBP < 140 mm Hg and average DBP < 90 mm Hg^11^.

Types of antihypertensive medications were classified into calcium channel blockers (CCBs), angiotensin-converting enzyme inhibitors (ACEIs)/angiotensin II receptor blockers (ARBs), β-blockers, diuretics, and others (including traditional Chinese medications, not guideline-recommended medications and medications with unknown components).

Overweight and obese were defined as a BMI between 24.0 and 27.9, and that of 28.0 or more, respectively, following the Chinese adults' guidelines for the prevention and control of overweight and obese^12^.

Non-smokers were referred to someone who had never smoked before the investigation. Past smokers were referred to someone who had a history of smoking cigarettes but had quit smoking for more than two weeks before the investigation. Current smokers were referred to someone who had a history of smoking cigarettes within two weeks before the study. And the rest of the involving information shares the exact definition as the protocol of this project^8^.

### Statistical analysis

The Continuous data are presented as the *mean* ± *Standard Deviation* (SD); statistical significance between the 2 groups was determined by *Student’s t-test*, and when among multiple groups, the *Kruskal–Wallis H test* was applied. Categorical data are presented as frequencies, percentages, and proportions. *Chi-squared tests* were used to compare differences among categorical variables. To compare with the national epidemiological data, the prevalence, awareness, treatment, control were age-standardized and sex-standardized to the national level, using data from the 2010 Population Census of the People's Republic of China^13^. To establish models for analyzing multiple risk factors of HTN for Han and Zhuang nationalities, we first used univariate logistic regression to confirm the significant related factors for HTN, variables with a *P* < 0.2 were selected into the multivariable logistic regression to analyze the multiple factors for HTN. The forward likelihood ratio (LR) method was used to adjust the model further. A *P* < 0.05 was considered as statistical significance. All statistical analysis was performed by SPSS 26.0 software (SPSS Inc., Chicago, USA) and R 4.0.5.

## Results

### Characteristics of participants

Our study recruited 17,100 participants. Due to missing essential information like the BP or other risk factors, 3287 of them were excluded. The retained cases used in the analysis were 13,813, including 6512 (47.1%) men and 7,301 (52.9%) women, 6506 (47.1%) were of Han nationality, 6923 (50.1%) were of Zhuang nationality, and 384 (2.8%) were of other minorities, the demographic characteristics are shown in Table 1. A significant difference was observed between men and women for all characteristics except for ethnicity, mean BMI, and family history of HTN. All characteristics except for age, sex, and history of alcohol consumption showed significant differences between Han and Zhuang nationalities.

**Table 1:** Characteristics of study group by sex and ethnicity.

Characteristics	Total	Ethnicity			*P* value for Han and Zhuang	Sex		*P* value for sex
		Han	Zhuang	Others		Men	Women	
N (%)	13,813 (100%)	6506 (47.1%)	6923 (50.1%)	384 (2.8%)		6512 (47.1%)	7301 (52.9%)	
Mean age (years)	46.87 ± 19.24	46.78 ± 18.99	47.43 ± 19.41	38.30 ± 18.47	0.051	46.05 ± 19.30	47.6 ± 19.17	< 0.001
Education level, n (%)*								
Never attended school	1628 (11.8%)	683 (10.5%)	926 (13.4%)	19 (4.9%)	< 0.001	351 (5.4%)	1277 (17.6%)	< 0.001
Elementary school	4245 (30.8%)	1930 (29.7%)	2162 (31.4%)	153 (39.8%)		1982 (30.5%)	2263 (31.1%)	
Elementary middle school	5895 (42.8%)	2909 (44.8%)	2793 (40.5%)	193 (50.3%)		3062 (47.1%)	2833 (38.9%)	
High school or above	2007 (14.6%)	977 (15.0%)	1011 (14.7%)	19 (4.9%)		1106 (17.0%)	901 (12.4%)	
Height (cm)	158.11 ± 8.62	159.03 ± 8.72	157.41 ± 8.47	155.03 ± 7.61	< 0.001	164.16 ± 6.57	152.7 ± 6.33	< 0.001
Weight (kg)	55.85 ± 9.69	57.06 ± 9.56	54.85 ± 9.68	53.38 ± 9.56	< 0.001	60.27 ± 9.13	51.9 ± 8.39	< 0.001
Mean BMI, kg/m^2^	22.29 ± 3.18	22.52 ± 3.14	22.08 ± 3.18	22.18 ± 3.48	< 0.001	22.35 ± 3.08	22.24 ± 3.26	0.058
Overweight or obesity: BMI ≥ 24 kg/m^2^, n (%)	3603 (26.1%)	1908 (29.3%)	1601 (23.1%)	94 (24.5%)	< 0.001	1757 (27.0%)	1846 (25.3%)	0.023
Overweight: 24 ≤ BMI < 28 kg/m^2^, n (%)	2981 (21.6%)	1599 (24.6%)	1311 (18.9%)	71 (18.5%)		1524 (23.4%)	1457 (20.0%)	
Obesity: BMI ≥ 28 kg/m^2^, n (%)	622 (4.5%)	309 (4.7%)	290 (4.2%)	23 (6.0%)		233 (3.6%)	389 (5.3%)	
Family history of HTN, n (%)	1185 (8.6%)	726 (11.2%)	439 (6.3%)	20 (5.2%)	< 0.001	552 (8.5%)	633 (8.7%)	0.685
**Smoking status**								
Non-smokers	11,522 (83.4%)	5480 (84.2%)	5714 (82.5%)	328 (85.4%)	0.002	4237 (65.1%)	7285 (99.8%)	< 0.001
Past smokers	156 (1.1%)	57 (0.9%)	97 (1.4%)	2 (0.5%)		152 (2.3%)	4 (0.1%)	
Current smokers	2135 (15.5%)	969 (14.9%)	1112 (16.1%)	54 (14.1%)		2123 (32.6%)	12 (0.2%)	
History of alcohol consumption, n (%)	2196 (15.9%)	1035 (15.9%)	1106 (16.0%)	55 (14.3%)	0.915	2099 (32.2%)	97 (1.3%)	< 0.001

*HTN* hypertension, *BMI* Body Mass Index.

Data are presented as n (%) or mean ± SD.

*Missing 38 cases.

### BP level and prevalence of HTN

**Figure 1:**
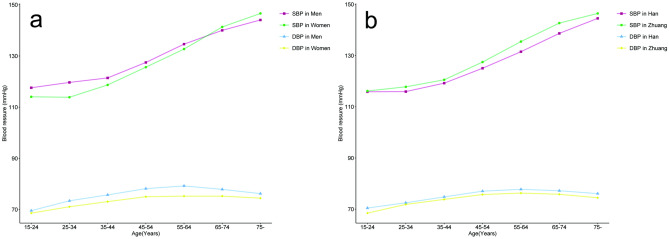
Blood pressure level by Sex and ethnicity. (**a**) By sex, (**b**) By ethnicity.

The mean SBP was 126.77 ± 19.10 mm Hg, and the mean DBP was 74.34 ± 9.76 mm Hg. Men, Zhuang nationality, overweight or obese, low education levels, smokers, and people with a history of alcohol consumption had higher mean SBP (*P* < 0.001 for all). Among all participants, 3014 were hypertensive, the crude prevalence of HTN for people aged ≥ 15 years in the Guangxi Zhuang Autonomous Region was 21.82%, the prevalence increased progressively with increased age (*P* < 0.001). Moreover, 5606 (40.58%) people in our study group were prehypertensive, and the majority of them were around middle age (35–64 years). There was no significant discrepancy of the crude prevalence between male and female participants (21.68% vs. 21.94%, *P* = 0.713), while Zhuang nationality had a higher prevalence than Han (23.50% vs. 20.35%, *P* < 0.001). Also, people with overweight or obesity, low education levels, a family history of HTN, smoking, and a history of alcohol consumption had a higher prevalence of HTN than their contrast (*P* < 0.05 for all, Table 2, Fig. 1).

**Table 2:** Blood pressure and prevalence of HTN by characteristics.

Characteristics	N	Blood Pressure (mmHg)			Hypertension classification, n (%)					prevalence of HTN, n (%)
		SBP	DBP	PP	Normal	Prehypertension	Stage 1	Stage 2	Stage 3	
Total	13,813	126.77 ± 19.10	74.34 ± 9.76	52.43 ± 15.63	5193 (37.60%)	5606 (40.58%)	1851 (13.40%)	750 (5.43%)	220 (1.59%)	3014 (21.82%)
**Age (years)**										
15–24	1787	115.87 ± 10.48	69.09 ± 7.60	46.79 ± 9.50	1137 (63.63%)	603 (33.74%)	45 (2.52%)	2 (0.11%)	0 (0.00%)	47 (2.63%)
25–34	2344	116.74 ± 10.49	72.23 ± 7.61	44.52 ± 9.05	1365 (58.23%)	917 (39.12%)	55 (2.35%)	7 (0.30%)	0 (0.00%)	62 (2.65%)
35–44	2606	119.97 ± 12.6	74.33 ± 8.12	45.65 ± 9.66	1178 (45.20%)	1252 (48.04%)	135 (5.18%)	27 (1.04%)	5 (0.19%)	176 (6.75%)
45–54	2171	126.46 ± 15.81	76.44 ± 9.86	50.02 ± 11.83	720 (33.16%)	1016 (46.80%)	322 (14.83%)	71 (3.27%)	13 (0.60%)	435 (20.04%)
55–64	1929	133.57 ± 19.47	77.02 ± 10.83	56.55 ± 15.07	430 (22.29%)	815 (42.25%)	439 (22.76%)	158 (8.19%)	35 (1.81%)	684 (35.46%)
65–74	1564	140.72 ± 21.44	76.47 ± 11.03	64.25 ± 17.53	224 (14.32%)	556 (35.55%)	439 (28.07%)	220 (14.07%)	69 (4.41%)	784 (50.13%)
≥ 75	1412	145.48 ± 22.67	75.23 ± 11.51	70.25 ± 19.26	139 (9.84%)	447 (31.66%)	416 (29.46%)	265 (18.77%)	98 (6.94%)	826 (58.50%)
*P* value		< 0.001	< 0.001	< 0.001		< 0.001				< 0.001
**Sex**										
Men	6512	127.5 ± 18.22	75.56 ± 9.70	51.94 ± 14.71	2157 (33.12%)	2943 (45.19%)	894 (13.73%)	334 (5.13%)	101 (1.55%)	1412 (21.68%)
Women	7301	126.12 ± 19.84	73.24 ± 9.68	52.88 ± 16.40	3036 (41.58%)	2663 (36.47%)	957 (13.11%)	416 (5.70%)	119 (1.63%)	1602 (21.94%)
*P* value		< 0.001	< 0.001	< 0.001		< 0.001				0.713
**Ethnicity**										
Han	6506	125.47 ± 18.29	74.98 ± 9.17	50.49 ± 15.21	2554 (39.26%)	2628 (40.39%)	823 (12.65%)	295 (4.53%)	87 (1.34%)	1324 (20.35%)
Zhuang	6923	128.21 ± 19.85	73.78 ± 10.22	54.43 ± 15.94	2467 (35.63%)	2829 (40.86%)	981 (14.17%)	440 (6.36%)	132 (1.91%)	1627 (23.50%)
Others	384	122.83 ± 16.68	73.5 ± 10.39	49.33 ± 12.12	172 (44.79%)	149 (38.80%)	47 (12.24%)	15 (3.91%)	1 (0.26%)	63 (16.41%)
*P* value for Han and Zhuang		< 0.001	< 0.001	< 0.001		< 0.001				< 0.001
**Body Mass Index (kg/m**^**2**^**)**										
< 24	10,210	124.7 ± 18.24	73.18 ± 9.3	51.51 ± 15.27	4312 (42.23%)	4088 (40.04%)	1128 (11.05%)	436 (4.27%)	139 (1.36%)	1810 (17.73%)
≥ 24	3603	132.65 ± 20.25	77.6 ± 10.28	55.05 ± 16.35	881 (24.45%)	1518 (42.13%)	723 (20.07%)	314 (8.71%)	81 (2.25%)	1204 (33.42%)
*P* value		< 0.001	< 0.001	< 0.001		< 0.001				< 0.001
Education level*										
Never attended school	1628	139.84 ± 22.34	75.21 ± 11.12	64.64 ± 18.95	275 (16.89%)	572 (35.14%)	418 (25.68%)	237 (14.56%)	75 (4.61%)	781 (47.97%)
Elementary school	4245	131.95 ± 20.4	75.83 ± 10.29	56.12 ± 16.88	1156 (27.23%)	1750 (41.22%)	822 (19.36%)	334 (7.87%)	101 (2.38%)	1339 (31.54%)
Elementary middle school	5895	121.79 ± 15.3	73.36 ± 9.13	48.43 ± 12.08	2688 (45.60%)	2502 (42.44%)	486 (8.24%)	136 (2.31%)	36 (0.61%)	705 (11.96%)
High school or above	2007	119.67 ± 14.47	73.35 ± 8.65	46.32 ± 10.88	1069 (53.26%)	762 (37.97%)	118 (5.88%)	39 (1.94%)	7 (0.35%)	176 (8.77%)
*P* value		< 0.001	< 0.001	< 0.001		< 0.001				< 0.001
**Family history of HTN**										
No family history	12,628	126.73 ± 19.16	74.2 ± 9.71	52.53 ± 15.79	4758 (37.68%)	5151 (40.79%)	1667 (13.20%)	689 (5.46%)	210 (1.66%)	2719 (21.53%)
Family history	1185	127.17 ± 18.45	75.8 ± 10.19	51.37 ± 13.83	435 (36.71%)	455 (38.40%)	184 (15.53%)	61 (5.15%)	10 (0.84%)	295 (24.89%)
*P* value		0.451	< 0.001	0.006		0.51				0.007
**Smoking status**										
Non-smokers	11,522	126.05 ± 18.95	73.89 ± 9.57	52.16 ± 15.61	4573 (39.69%)	4545 (39.45%)	1471 (12.77%)	601 (5.22%)	171 (1.48%)	2404 (20.86%)
Past smokers	156	140.69 ± 24.01	78.62 ± 12.20	62.07 ± 19.42	32 (20.51%)	48 (30.77%)	35 (22.44%)	26 (16.67%)	10 (6.41%)	76 (48.72%)
Current smokers	2135	129.65 ± 18.89	76.45 ± 10.20	53.20 ± 15.22	588 (27.54%)	1013 (47.45%)	345 (16.16%)	123 (5.76%)	39 (1.83%)	534 (25.01%)
*P* value		< 0.001	< 0.001	< 0.001		< 0.001				< 0.001
**History of alcohol consumption**										
No history of alcohol consumption	11,617	126.09 ± 19.01	73.75 ± 9.56	52.34 ± 15.71	4612 (39.70%)	4592 (39.53%)	1480 (12.74%)	596 (5.13%)	180 (1.55%)	2413 (20.77%)
With an alcohol-consuming history	2196	130.38 ± 19.22	77.43 ± 10.23	52.95 ± 15.22	581 (26.46%)	1014 (46.17%)	371 (16.89%)	154 (7.01%)	40 (1.82%)	601 (27.37%)
*P* value		< 0.001	< 0.001	0.084		< 0.001				< 0.001

*HTN* hypertension, *BMI* Body Mass Index, *SBP* Systolic Blood Press, *DBP* Diastolic Blood Pressure, *PP* Pulse Pressure.

Data are presented as n (%) or mean ± SD.

*Missing 38 cases.

We gave different weights to age and sex-specific subgroups to match the distribution according to the census data to compare with the national level. The age-standardized and sex-standardized overall prevalence was 15.80%, and the age-standardized prevalence for men was 16.59%, higher than that for women (14.96%, Table 3).

**Table 3:** Age and sex-standardized prevalence, awareness, treatment, control of HTN for the study group, and age-standardized rates for men and women.

	Total (%)	Men (%)	Women (%)
Prevalence	15.80	16.59	14.96
Awareness	16.48	15.22	17.76
Treatment	11.99	10.11	13.91
Control	3.62	3.03	4.22
Control among treated HTN patients	23.56	17.98	16.33

Data are presented as n%; the standardized procedure was according to the data of the 2010 Population Census of the People's Republic of China.

The prevalence of HTN was even approximately twice higher when using the 2017 ACC/AHA criteria (Table 4).

**Table 4:** Prevalence and control rate of HTN based on 2010 Chinese high blood pressure guideline and 2017 ACC/AHA high blood pressure guideline.

Characteristics	Prevalence, n (%)		Control, n (%)		Control among treated HTN participants, n (%)	
	2010 Chinese	2017 ACC/AHA	2010 Chinese	2017 ACC/AHA	2010 Chinese	2017 ACC/AHA
Total	3014 (21.82%)	5967 (43.20%)	193 (6.40%)	74 (1.24%)	193 (25.03%)	74 (9.60%)
**Age (years)**						
15–24	47 (2.63%)	247 (13.82%)	0 (0.00%)	0 (0.00%)	0 (0.00%)	0 (0.00%)
25–34	62 (2.65%)	475 (20.26%)	0 (0.00%)	0 (0.00%)	0 (0.00%)	0 (0.00%)
35–44	176 (6.75%)	758 (29.09%)	9 (5.11%)	5 (0.66%)	9 (42.86%)	5 (23.81%)
45–54	435 (20.04%)	1023 (47.12%)	29 (6.67%)	14 (1.37%)	29 (35.37%)	14 (17.07%)
55–64	684 (35.46%)	1186 (61.48%)	52 (7.60%)	17 (1.40%)	52 (27.37%)	17 (8.95%)
65–74	784 (50.13%)	1157 (73.98%)	56 (7.14%)	22 (1.90%)	56 (23.93%)	22 (9.40%)
≥ 75	826 (58.50%)	1121 (79.39%)	47 (5.69%)	16 (1.43%)	47 (19.26%)	16 (6.56%)
*P* value	< 0.001	< 0.001	0.087	0.013	0.01	0.012
**Sex**						
Men	1412 (21.68%)	3001 (46.08%)	83 (5.88%)	30 (1.00%)	83 (26.69%)	30 (9.65%)
Women	1602 (21.94%)	2966 (40.62%)	110 (6.87%)	44 (1.48%)	110 (23.91%)	44 (9.57%)
*P* value	0.713	< 0.001	0.269	0.091	0.383	0.97
**Ethnicity**						
Han	1324 (20.35%)	2789 (42.87%)	119 (8.99%)	46 (1.65%)	119 (29.38%)	46 (11.36%)
Zhuang	1627 (23.50%)	3042 (43.94%)	74 (4.55%)	28 (0.92%)	74 (20.33%)	28 (7.69%)
Others	63 (16.41%)	136 (35.42%)	0 (0.00%)	0 (0.00%)	0 (0.00%)	0 (0.00%)
*P* value for Han and Zhuang	< 0.001	0.21	< 0.001	0.013	0.004	0.085
**Body Mass Index (kg/m**^**2**^**)**						
< 24	1810 (17.73%)	3828 (37.49%)	107 (5.91%)	43 (1.12%)	107 (26.35%)	43 (10.59%)
≥ 24	1204 (33.42%)	2139 (59.37%)	86 (7.14%)	31 (1.45%)	86 (23.56%)	31 (8.49%)
*P* value	< 0.001	< 0.001	0.176	0.275	0.371	0.323
Education level*						
Never attended school	781 (47.97%)	1127 (69.23%)	51 (6.53%)	18 (1.60%)	51 (22.77%)	18 (8.04%)
Elementary school	1339 (31.54%)	2399 (56.51%)	82 (6.12%)	32 (1.33%)	82 (23.63%)	32 (9.22%)
Elementary middle school	705 (11.96%)	1895 (32.15%)	47 (6.67%)	18 (0.95%)	47 (30.13%)	18 (11.54%)
High school or above	176 (8.77%)	524 (26.11%)	12 (6.82%)	5 (0.95%)	12 (30.00)	5 (12.50%)
*P* value	< 0.001	< 0.001	0.955	0.388	0.301	0.624
**Family history of HTN**						
No family history	2719 (21.53%)	5404 (42.79%)	153 (5.63%)	61 (1.13%)	153 (24.09%)	61 (9.61%)
Family history	295 (24.89%)	563 (47.51%)	40 (13.56%)	13 (2.31%)	40 (29.41%)	13 (9.56%)
*P* value	0.007	0.002	< 0.001	0.016	0.194	0.986
**Smoking status**						
Non-smokers	2404 (20.86%)	4747 (41.20%)	161 (6.70%)	63 (1.33%)	161 (25.00%)	63 (9.78%)
Past smokers	76 (48.72%)	108 (69.23%)	5 (6.58%)	1 (0.93%)	5 (19.23%)	1 (3.85%)
Current smokers	534 (25.01%)	1112 (52.08%)	27 (5.06%)	10 (0.90%)	27 (26.73%)	10 (9.90%)
*P* value	< 0.001	< 0.001	0.374	0.488	0.733	0.598
**History of alcohol consumption**						
No history of alcohol consumption	2413 (20.77%)	4776 (41.11%)	157 (6.51%)	65 (1.36%)	157 (24.08%)	65 (9.97%)
With an alcohol-consuming history	601 (27.37%)	1191 (54.23%)	36 (5.99%)	9 (0.76%)	36 (30.25%)	9 (7.56%)
*P* value	< 0.001	< 0.001	0.644	0.091	0.153	0.413

*HTN* Hypertension, *BMI* Body Mass Index.

Data are presented as n (%).

*Missing 38 cases.

### Awareness, treatment, control of HTN

People who had a clear awareness about their BP status were 1005 (33.34%), 771 (25.58%) who were taking antihypertensive agents in the past two weeks, and 193 (6.40%) who got the appropriate control of their BP. Moreover, among people with treated HTN, only 25.03% were controlled.

Older people had higher awareness and treatment rates of HTN (*P* < 0.001 for all), but the control rate of BP was not significantly different from the youngers (*P* = 0.087); among those people with treated HTN, mid-aged (35–64 years) people tended to acquire better control of their BP. Crude rates of awareness and treatment for women were higher than those for men (35.89% vs. 30.45%, 28.71% vs. 22.03%, respectively, *P* < 0.05 for all), However, the rate of control and the efficiency of treatment were similar. People of Han nationality had better awareness, treatment, control rates of HTN than those of Zhuang (37.39% vs. 31.22%, 30.59% vs. 22.37%, 8.99% vs. 4.55%, respectively, *P* < 0.05 for all), Moreover, the efficiency of treatment in Han was significantly higher than that of Zhuang (29.38% vs. 20.33%, *P* < 0.05). People with overweight or obesity, or a lower education level, had higher awareness and treatment rates, but the control rate was not significantly different. People with a family history of HTN showed better awareness, treatment, and control of HTN than others. Past smokers tended to be aware of HTN more and were willing to get treatment more than others, but the control rate of HTN showed no difference from that of others. People with a history of alcohol consumption had a higher treatment, while the awareness and control of HTN were not different from others (Table 5).

**Table 5:** Awareness, treatment, and control of HTN by characteristics.

Characteristics	Hypertension, n	Awareness, n (%)	Treatment, n (%)	Control, n (%)	Control among treated HTN participants, n (%)
Total	3014	1005 (33.34%)	771 (25.58%)	193 (6.40%)	193 (25.03%)
**Age (years)**					
15–24	47	0 (0.00%)	0 (0.00%)	0 (0.00%)	0 (0.00%)
25–34	62	2 (3.23%)	0 (0.00%)	0 (0.00%)	0 (0.00%)
35–44	176	31 (17.61%)	21 (11.93%)	9 (5.11%)	9 (42.86%)
45–54	435	113 (25.98%)	82 (18.85%)	29 (6.67%)	29 (35.37%)
55–64	684	242 (35.38%)	190 (27.78%)	52 (7.60%)	52 (27.37%)
65–74	784	295 (37.63%)	234 (29.85%)	56 (7.14%)	56 (23.93%)
≥ 75	826	322 (38.98%)	244 (29.54%)	47 (5.69%)	47 (19.26%)
*P* value		< 0.001	< 0.001	0.087	0.01
**Sex**					
Men	1412	430 (30.45%)	311 (22.03%)	83 (5.88%)	83 (26.69%)
Women	1602	575 (35.89%)	460 (28.71%)	110 (6.87%)	110 (23.91%)
*P* value		0.002	< 0.001	0.269	0.383
**Ethnicity**					
Han	1324	495 (37.39%)	405 (30.59%)	119 (8.99%)	119 (29.38%)
Zhuang	1627	508 (31.22%)	364 (22.37%)	74 (4.55%)	74 (20.33%)
Others	63	2 (3.17%)	2 (3.17%)	0 (0.00%)	0 (0.00%)
*P* value for Han and Zhuang		< 0.001	< 0.001	< 0.001	0.004
**Body Mass Index (kg/m**^**2**^**)**					
< 24	1810	542 (29.94%)	406 (22.43%)	107 (5.91%)	107 (26.35%)
≥ 24	1204	463 (38.46%)	365 (30.32%)	86 (7.14%)	86 (23.56%)
*P* value		< 0.001	< 0.001	0.176	0.371
Education level*					
Never attended school	781	293 (37.52%)	224 (28.68%)	51 (6.53%)	51 (22.77%)
Elementary school	1339	442 (33.01%)	347 (25.91%)	82 (6.12%)	82 (23.63%)
Elementary middle school	705	207 (29.36%)	156 (22.13%)	47 (6.67%)	47 (30.13%)
High school or above	176	58 (32.95%)	40 (22.73%)	12 (6.82%)	12 (30.00)
*P* value		0.011	0.027	0.955	0.301
**Family history of HTN**					
No family history	2719	838 (30.82%)	635 (23.35%)	153 (5.63%)	153 (24.09%)
Family history	295	167 (56.61%)	136 (46.10%)	40 (13.56%)	40 (29.41%)
*P* value		< 0.001	< 0.001	< 0.001	0.194
**Smoking status**					
Non-smokers	2404	822 (34.19%)	644 (26.79%)	161 (6.70%)	161 (25.00%)
Past smokers	76	34 (44.74%)	26 (34.21%)	5 (6.58%)	5 (19.23%)
Current smokers	534	149 (27.90%)	101 (18.91%)	27 (5.06%)	27 (26.73%)
*P* value		0.002	< 0.001	0.374	0.733
**History of alcohol consumption**					
No history of alcohol consumption	2413	823 (34.11%)	652 (27.02%)	157 (6.51%)	157 (24.08%)
With an alcohol-consuming history	601	182 (30.28%)	119 (19.80%)	36 (5.99%)	36 (30.25%)
*P* value		0.075	< 0.001	0.644	0.153

*HTN* Hypertension, *BMI* Body Mass Index.

Data are presented as n (%).

*Missing 38 cases.

The age and sex-standardized awareness, treatment, and control rates of HTN were 16.48%, 11.99%, 3.62%, respectively, and the standardized control rate among treated HTN was 23.56%. The age-standardized awareness, treatment, and control rate of HTN for women were all higher than those for men (17.76% vs. 15.22%, 13.91% vs. 10.11%, 4.22% vs. 3.03%, respectively), but the control rate of treated HTN for women was lower than that for men (16.33%vs. 17.98%, Table 3).

### The usage of antihypertensive medications

The most common antihypertensive strategy in our study was using a single type of medication like CCBs or ACEIs/ARBs. And a considerable proportion of them was using some unusual medications, including traditional Chinese medicines, Non-guideline-recommended drugs, and medicines with unknown components (Table 6).

**Table 6:** The usage of antihypertensive medications in the study group.

Medication type	Total (n = 771)	Han (n = 405)	Zhuang (n = 364)	Men (n = 311)	Women (n = 460)
**Mono-pill therapy**					
CCBs	318 (41.25%)	192 (47.41%)	125 (34.34%)	129 (41.48%)	189 (41.09%)
ACEIs or ARBs	82 (10.64%)	35 (8.64%)	47 (12.91%)	32 (10.29%)	50 (10.87%)
β-blockers	26 (3.37%)	3 (0.74%)	22 (6.04%)	15 (4.82%)	11 (2.39%)
Diuretics	12 (1.56%)	8 (1.98%)	4 (1.10%)	7 (2.25%)	5 (1.09%)
**Single-pill combinations**					
CCBs & ACEIs or ARBs	22 (2.85%)	10 (2.47%)	12 (3.30%)	7 (2.25%)	15 (3.26%)
CCBs & β-blockers	8 (1.04%)	4 (0.99%)	4 (1.10%)	4 (1.29%)	4 (0.87%)
CCBs & Diuretics	5 (0.65%)	3 (0.74%)	2 (0.55%)	3 (0.96%)	2 (0.43%)
ACEIs or ARBs & β-blockers	7 (0.91%)	1 (0.25%)	6 (1.65%)	5 (1.61%)	2 (0.43%)
ACEIs or ARBs & diuretics	2 (0.26%)	1 (0.25%)	1 (0.27%)	0 (0.00%)	2 (0.43%)
β-blockers & diuretics	1 (0.13%)	0 (0.00%)	1 (0.27%)	0 (0.00%)	1 (0.22%)
Triple-pill combinations	3 (0.39%)	3 (0.74%)	0 (0.00%)	2 (0.64%)	1 (0.22%)
Others*	285 (36.96%)	145 (35.80%)	140 (38.46%)	107 (34.41%)	178 (38.70%)
P value		< 0.001		0.272	

*CCBs* Calcium Channel Blockers, *ACEIs* Angiotensin-Converting Enzyme Inhibitors, *ARBs* Angiotensin II Receptor Blockers.

Data are presented as n (%).

*Including traditional Chinese medications, not guideline-recommended medications, and medications with unknown components.

### Multivariable logistic regression analysis for HTN

**Figure 2:**
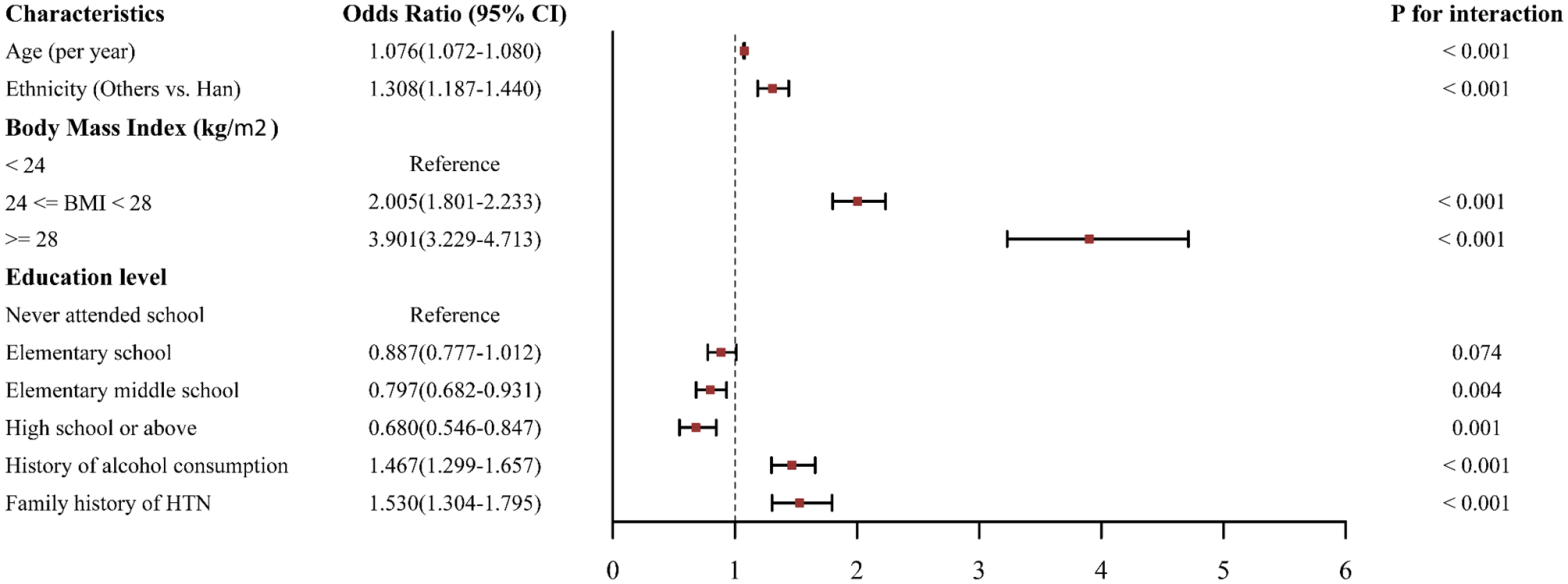
Multiple factors of HTN for the study group.

The univariate logistic regression analysis showed that age, non-Han nationalities, overweight or obesity, low education levels, a family history of HTN, smokers, and the history of alcohol consumption were significantly related to HTN (*P* < 0.05 for all), while sex was not a significantly related factor in our study group. (Supplemental Table S1) Adjusted multivariable logistic regression model further confirmed that age, non-Han nationalities, overweight or obesity, a family history of HTN, and a history of alcohol consumption were positively related to HTN in the whole group, while higher education level was negative. (Supplemental Table S1–2, Fig. 2).

**Figure 3:**
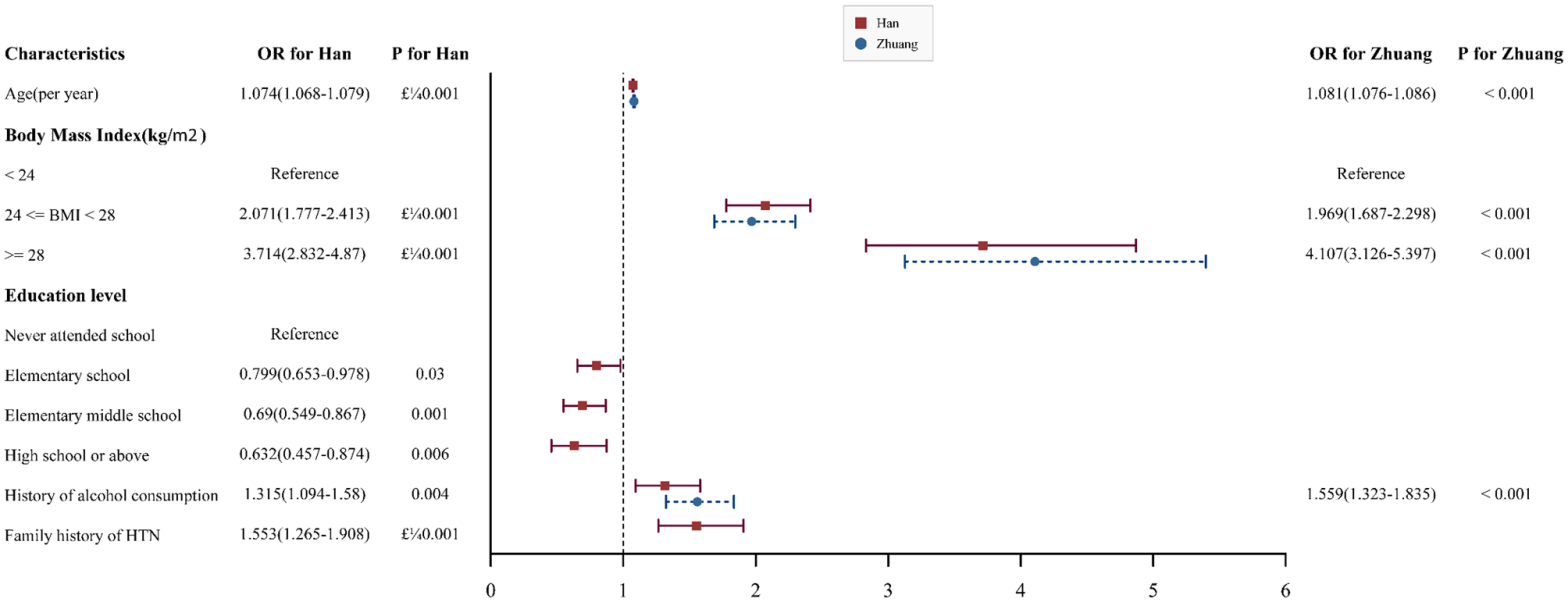
Risk factors of HTN for the Han and Zhuang nationalities.

All significantly related factors of HTN for the Han and Zhuang nationalities were the same except for the current smoking state and family history of HTN; as current smoking was positively related to Zhuang nationality and not to Han, then the family history was significantly associated with Han nationality and not with Zhuang. And the adjusted multivariable logistic regression model for Han nationality showed age, overweight or obesity, a family history of HTN, and a history of alcohol consumption were positively related factors for HTN, while higher education levels were negative. Moreover, the adjusted multivariable logistic regression model for Zhuang nationality showed that age, overweight or obesity, the history of alcohol consumption were significant related factors. (Supplemental Table S1, 3–4, Fig. 3).

## Discussion

In our study, 17,100 residents were enrolled in representing the epidemiological status of HTN of the population age ≥ 15 years in Guangxi Zhuang Autonomous Region, and it was the first to include such a large number of samples in Guangxi. The investigation revealed that the age and sex-standardized prevalence of HTN was 15.80%. Among the people with HTN, the standardized rates of awareness, treatment, and control were extremely low (16.48%, 11.99%, 3.62%, respectively); and less than a quarter of treated HTN were controlled. The crude rate of prevalence was twice higher when using the 2017 ACC/AHA guideline criteria, and the control rate was only 1.24%.

The epidemiological status of HTN in China varied in different phases of time. A nationwide study that started in 1991 and included 950,356 participants showed that the prevalence of HTN was 13.6% among people aged 15 years or older; in 2002, the China Nutrition and Health survey released a result that approximately 153 million people (accounted for 18%) of the population aged 18 or older were hypertensive. A study with a large population nationwide during 2009–2010 suggested that the increasing prevalence was 29.6%^14–16^. The prevalence of HTN also diverged in multiple phases of time in Guangxi. A sampling survey recruited dwellers from 4 cities found the prevalence of HTN was 15.3% among dwellers aged 15 years or more in 2007. Another sampling survey containing 3,363 samples showed that the prevalence of HTN was 13.45% for people aged 15 or older from 7 areas in 2009^6,7^. In our study, the standardized prevalence of HTN in Guangxi was lower than the national level in the present study (15.80% vs. 23.2%)^17^, it was higher than those shown in previous studies about HTN in Guangxi. It suggested that the hypertensive population in Guangxi had less proportion than the whole nation, and HTN was becoming more prevalent.

This study indicated that older men, Zhuang nationality, low education level, overweight or obese, smokers, and people with a history of alcohol consumption had higher mean SBP than others. The prevalence of HTN among age 45 or older, Zhuang nationality, low education levels, overweight or obesity, with a family history of HTN, smokers, and people with a history of alcohol consumption were significantly higher. However, the crude prevalence showed no significant difference between men and women, which was inconsistent with previous studies^6,7,15–17^. That was probably because of the older mean age for women than men in this study (47.6 ± 19.17 years vs. 46.05 ± 19.30 years, *P* < 0.05), when age-standardized, the prevalence of men was higher than that of women (16.59% vs. 14.96%). The adjusted multivariable logistic regression model indicated that older age, non-Han nationalities, overweight or obese, a family history of HTN, and a history of alcohol consumption were positively associated with HTN, while higher educational levels were negative, some of which were consistent with some other studies^15–17^. The smoking status was different between BTN and people with a normal BP, and it was also significantly related to HTN in the univariate logistic regression analysis; past smokers and current smokers were at higher risk of HTN than non-smokers. However, when adjusted for all confounders, it was not significantly associated with HTN in the current study. Interestingly, The fractions of people overweight or obese, with a family history of HTN, and smokers in our study were lower than the national level in the present study, which could be some of the explanations for its lower prevalence of HTN in Guangxi^17^.

Our study also showed remarkable heterogeneities of the BP status between Han and Zhuang nationality; both mean SBP and prevalence of HTN in Zhuang were higher than those in Han, and the distribution of age or sex had no discrepancy for those two. The previous study showed that BP levels and prevalence of HTN were various among different ethnicities in China^18^, due to the lack of prior large-scale surveys, we could only address our results to some previous small-scale studies. They also found that Zhuang nationality had a higher SBP level and prevalence of HTN; risk factors of HTN were different for Han and Zhuang^19–21^. Analysis of multiple factors for HTN in this study showed that age, overweight or obese, and a history of alcohol consumption were positively associated with both Han and Zhuang nationalities. Education levels and the family history of HTN were also related to Han while they were significant factors for Zhuang when the model was adjusted for all confounders. Notably, older age, overweight or obese, and a history of alcohol consumption were consistently associated with a higher risk of HTN regardless of different nationalities.

The extremely low awareness rate of HTN in our study drew a big concern, it was a lot lower than the national level (16.48% vs. 46.9%), and it was not increasing compared with the prior studies. The treatment and control rates of HTN were also adverse. The treatment rate was not improving than those in the past, and it was still far lower than the national level (11.99% vs. 40.7%). The control of NTH was inadequate (3.62% vs. 15.3%). Even for those taking treatment of HTN, very few of them had their BP in a reasonable control; the treatment was not sufficient^6,7,17^. The analysis of treatment strategies for HTN in this study showed that a considerable proportion of people was using unusual antihypertensive medications, and even for those using guideline-recommended drugs, most of them were just under mono-type of pill therapy, which was not complying with the guideline; these could partially be related to the inefficiency of the antihypertensive therapy. Our study found that aged people tended to pay more attention to their BP status and get more treatment of HTN, but control rates of HTN among different age strata were not significantly different. People who were overweight or obese, had a family history of HTN, were past smokers had higher awareness rates, and seemed willing to take more treatment than others, but the control rate also showed no discrepancy. Women had higher awareness and treatment of HTN than men, and the age-standardized rates were also higher; however, the control rate had no dissimilarity. To note, the awareness, treatment, and control rates of HTN were all significantly higher in the Han nationality than in Zhuang. Our study also found that awareness and treatment in people with lower education levels were heightened, contradicting some other studies^17,22^. The possible explanation could be that the lower education population in our study had the older mean age; moreover, most of them were women. When we performed logistic regress analysis and adjusted the age and sex confounders to analyze the association between awareness of HTN and education levels, we found that education levels were not significantly associated with the awareness of HTN in the current study (P > 0.05, Supplemental Table S5). The control rate of HTN was again not significantly different among various education levels.

Aging is a well-established contributor to the development of HTN^23^; our study was also consistent with it, and with the future trend of aging, urbanization for the population in Guangxi, the prevalence of HTN was considered to keep increasing. Meanwhile, the not-improving and low awareness, treatment, and control rates of HTN would make the HTN a much more severe public health problem for Guangxi. The challenge for better management of HTN was imminent. Our study showed that overweight or obese, the history of alcohol consumption were risk factors of HTN for both Han and Zhuang nationalities, and they were consistent when all confounders were adjusted. These two modifiable risk factors suggested that strategies to intervene in HTN can be to keep a fit BMI and to quit alcohol. Albeit, better treatment, and BP control still need further study to explore solutions. The difference between Han and Zhuang nationality also needs deeper surveys to clarify, and region-specific strategies are required.

However, there are some limitations to our study. The study group's dietary data were not collected, and some dietary habits were considered to be related to BP. The blood testing data were not measured in the whole population, some of which were shown to have effects on BP^19–21^. These limitations might result in some undetected possible risk factors of HTN. Moreover, due to the unique structure of ethnicities in Guangxi, the epidemiological features of HTN can also be very different. It may result in some risk factors that are not discovered in our study. In addition, we got 3,287 samples excluded for missing important information, which can lead to some bias. Fortunately, the missing cases are stochastic, and the sample size in our study is large. The bias can be limited to a certain level. Further studies about HTN in Guangxi need to be performed.

## Conclusions

The prevalence of HTN in the population age ≥ 15 of the Guangxi Zhuang Autonomous Region was lower than the national level and increasing. The awareness, treatment, and control rates were deficient and not improving. Zhuang nationality had a higher prevalence of HTN, while the awareness, treatment and control rates were lower than Han's. Paying attention to the fit BMI and non-alcohol life style might be helpful strategies to intervene in HTN. More region-specific and efficient strategies for the control of HTN need to be enacted.

## Supplementary Information

Supplementary Information.
