# Supplementary material for: Prevalence, awareness, treatment and control of hypertension in Guangxi Zhuang Autonomous Region
Source: Sci Rep. 2022 Jan 18;12:900. doi: 10.1038/s41598-021-04735-1 (PMC8766488; doi:10.1038/s41598-021-04735-1)
Supplement: Supplementary file 1 — Supplementary Information. [file 41598_2021_4735_MOESM1_ESM.docx]

DATA SUPPLEMENT

Supplemental Table S1. Univariate logistic regression analysis of HTN for the study group

| Characteristics | OR(95% CI) for overall | P for overall | OR(95% CI) for Han | P for Han | OR(95% CI) for Zhuang | P for Zhuang |
| --- | --- | --- | --- | --- | --- | --- |
| Age (per year) | 1.076(1.073-1.079) | ＜0.001 | 1.076(1.071-1.081) | ＜0.001 | 1.077(1.073-1.082) | ＜0.001 |
| Sex(Woman vs. Man) | 1.015(0.936-1.101) | 0.713 | 1.076(0.953-1.214) | 0.236 | 0.975(0.872-1.09) | 0.656 |
| Ethnicity (Others vs. Han) | 1.178(1.086-1.277) | ＜0.001 |  |  |  |  |
| BMI (kg/㎡) |  |  |  |  |  |  |
| ＜24 | Reference |  | Reference |  | Reference |  |
| 24≤BMI＜28 | 2.114(1.927-2.319) | ＜0.001 | 2.425(2.123-2.771) | ＜0.001 | 1.937(1.695-2.214) | ＜0.001 |
| ≥28 | 3.583(3.034-4.232) | ＜0.001 | 4.064(3.199-5.163) | ＜0.001 | 3.236(2.543-4.118) | ＜0.001 |
| History of alcohol consumption | 1.437(1.295-1.595) | ＜0.001 | 1.238(1.056-1.451) | 0.008 | 1.62(1.407-1.867) | ＜0.001 |
| Smoking status |  |  |  |  |  |  |
| Non-smokers | Reference |  | Reference |  | Reference |  |
| Past smokers | 3.603(2.624-4.948) | ＜0.001 | 3.637(2.153-6.143) | ＜0.001 | 3.579(2.392-5.355) | ＜0.001 |
| Current smokers | 1.265(1.136-1.409) | ＜0.001 | 1.118(0.947-1.321) | 0.189 | 1.355(1.172-1.567) | ＜0.001 |
| Education level |  |  |  |  |  |  |
| Never attended school | Reference |  | Reference |  | Reference |  |
| Elementary school | 0.5(0.445-0.562) | ＜0.001 | 0.42(0.351-0.503) | ＜0.001 | 0.579(0.495-0.677) | ＜0.001 |
| Elementary middle school | 0.147(0.13-0.167) | ＜0.001 | 0.136(0.113-0.164) | ＜0.001 | 0.159(0.134-0.188) | ＜0.001 |
| High school or above | 0.104(0.087-0.125) | ＜0.001 | 0.091(0.069-0.12) | ＜0.001 | 0.115(0.089-0.147) | ＜0.001 |
| Family history of HTN | 1.208(1.052-1.387) | 0.007 | 1.347(1.125-1.613) | 0.001 | 1.123(0.9-1.403) | 0.305 |

Abbreviation,

HTN = hypertension; BMI = Body Mass Index; OR = Odds Ratio; CI = Confidential Interval

Supplemental Table S2. Multiple factors of HTN for the study group

| Characteristics | OR(95% CI) | P value |
| --- | --- | --- |
| Age (per year) | 1.076(1.072-1.080) | ＜0.001 |
| Ethnicity (Others vs. Han) | 1.308(1.187-1.440) | ＜0.001 |
| BMI (kg/㎡) |  |  |
| ＜24 | Reference |  |
| 24≤BMI＜28 | 2.005(1.801-2.233) | ＜0.001 |
| ≥28 | 3.901(3.229-4.713) | ＜0.001 |
| Education level |  |  |
| Never attended school | Reference |  |
| Elementary school | 0.887(0.777-1.012) | 0.074 |
| Elementary middle school | 0.797(0.682-0.931) | 0.004 |
| High school or above | 0.680(0.546-0.847) | 0.001 |
| History of alcohol consumption | 1.467(1.299-1.657) | ＜0.001 |
| Family history of HTN | 1.530(1.304-1.795) | ＜0.001 |

Abbreviation,

HTN = hypertension; BMI = Body Mass Index; OR = Odds Ratio; CI = Confidential Interval

Supplemental Table S3. Multiple factors of HTN for the Han nationality

| Characteristics | OR(95% CI) | P value |
| --- | --- | --- |
| Age (per year) | 1.074(1.068-1.079) | ＜0.001 |
| BMI (kg/㎡) |  |  |
| ＜24 | Reference |  |
| 24≤BMI＜28 | 2.071(1.777-2.413) | ＜0.001 |
| ≥28 | 3.714(2.832-4.87) | ＜0.001 |
| Education level |  |  |
| Never attended school | Reference |  |
| Elementary school | 0.799(0.653-0.978) | 0.030 |
| Elementary middle school | 0.69(0.549-0.867) | 0.001 |
| High school or above | 0.632(0.457-0.874) | 0.006 |
| History of alcohol consumption | 1.315(1.094-1.58) | 0.004 |
| Family history of HTN | 1.553(1.265-1.908) | ＜0.001 |

Abbreviation,

HTN = hypertension; BMI = Body Mass Index; OR = Odds Ratio; CI = Confidential Interval

Supplemental Table S4. Multiple factors of HTN for the Zhuang nationality

| Characteristics | OR(95% CI) | P value |
| --- | --- | --- |
| Age (per year) | 1.081(1.076-1.086) | ＜0.001 |
| BMI (kg/㎡) |  |  |
| ＜24 | Reference |  |
| 24≤BMI＜28 | 1.969(1.687-2.298) | ＜0.001 |
| ≥28 | 4.107(3.126-5.397) | ＜0.001 |
| History of alcohol consumption | 1.559(1.323-1.835) | ＜0.001 |

Abbreviation,

HTN = hypertension; BMI = Body Mass Index; OR = Odds Ratio; CI = Confidential Interval

Supplemental Table S5. Association between education levels and awareness of HTN for the study group

| Characters | OR(95% CI) | P value |
| --- | --- | --- |
| Unadjusted |  |  |
| Never attended school | Reference |  |
| Elementary school | 0.53(0.451-0.622) | ＜0.001 |
| Elementary middle school | 0.166(0.137-0.2) | ＜0.001 |
| High school or above | 0.136(0.101-0.181) | ＜0.001 |
| Adjusted for age and sex |  |  |
| Never attended school | Reference |  |
| Elementary school | 1.033(0.864-1.234) | 0.724 |
| Elementary middle school | 1.061(0.842-1.338) | 0.614 |
| High school or above | 1.187(0.848-1.661) | 0.319 |

Abbreviation,

HTN = hypertension; OR = Odds Ratio; CI = Confidential Interval
